# Cannabis Use Among Cancer Patients During Active Treatment: Findings From a Study at an NCI‐Designated Cancer Center

**DOI:** 10.1002/cam4.70384

**Published:** 2024-11-02

**Authors:** Amrit Baral, Bria‐Necole A. Diggs, Ranya Marrakchi El Fellah, Connor McCarley, Frank Penedo, Claudia Martinez, Denise C. Vidot

**Affiliations:** ^1^ Miller School of Medicine University of Miami Miami Florida USA; ^2^ Sylvester Comprehensive Cancer Center University of Miami Miami Florida USA; ^3^ School of Nursing and Health Studies University of Miami Coral Gables Florida USA

**Keywords:** cancer, cancer treatment chemotherapy, cannabis, CBD, immunotherapy, marijuana, radiation, THC

## Abstract

**Objective:**

This study aims to describe patterns, sources, and reasons for cannabis use among cancer patients during active treatment (+CDTX) compared to no‐use during active treatment (−CDTX).

**Methods:**

Data are from 385 surveys collected via REDCap during phase I of an ongoing study among adult cancer patients seen at an NCI‐designated comprehensive cancer center within the last 5 years of treatment. A harmonized survey was created with 11 other NCI centers to assess cannabis use patterns, sources, and reasons for use. Sociodemographics and cancer details were also collected via self‐report. Descriptive statistics were calculated and stratified by +/−CDTX. Chi‐squared tests were conducted to compare proportions between groups.

**Results:**

Among the sample [49.5 years (SD 15.9); 53.0% male; and 41.6% Hispanic/Latino], 41.0% + CDTX and 59.0% −CDTX. A majority (71.8%) of +CDTX initiated use before diagnosis versus 44.1% in −CDTX (*p* < 0.0001); patients diagnosed with stage 4 cancer had a statistically significant higher prevalence of +CDTX (60.0%; *p* = 0.003); 53.3% in radiation reported +CDTX compared to 42.8% in chemotherapy, and 36.4% in immunotherapy. Dispensaries and local dealers were the top sources of cannabis in both groups. Among +CDTX, 44.3% consumed cannabis at least once a day DTX, dominant cannabinoids used were CBD (35.2%), Delta‐8‐THC (18.3%), and CBD + THC ratio (14.1%); 12.7% were unsure what they consumed. Joints were the most common inhalation method (61.5%), and store‐bought candy was the most common edible (39.2%). Depression/mood, pain, and enjoyment were the top three reasons for +CDTX compared to enjoyment, depression/mood, and nausea/upset stomach in −CDTX (*p* = 0.02).

**Conclusions:**

Patterns, sources, and reasons for cannabis use varied between +CDTX and ‐CDTX. Future studies should examine the impacts of cannabis and specific cannabinoids on cancer treatment, drug interactions, survival outcomes, and quality of life.

## 1 Introduction

Cannabis use among cancer patients has gained significant attention due to its potential therapeutic benefits in managing symptoms associated with cancer and the adverse effects of various treatment modalities [1]. The patterns and reasons for cannabis use during active and post‐treatment cycles vary widely, influenced by factors such as side effects, prior exposure, stigmas, and sociodemographic characteristics [2]. With the increasing legalization, normalization, and availability of various cannabis products, understanding its usage patterns among cancer patients undergoing active treatment has become critically important [3]. In the United States, expanding legal protections for medical cannabis has led both cancer care teams and patients to consider integrating cannabis as an adjunct to active and post‐treatment regimens to enhance pain management, reduce stress and nausea, and improve sleep patterns [4]. As of November 2023, cannabis is legally available for medical use in 38 states, three territories, and Washington, DC, and for recreational use in 24 states, two territories, and Washington, DC [5]. Despite this growing interest, there remains a notable gap in the literature regarding the specific patterns, sources, and reasons for cannabis use among cancer patients under active treatments.

Active cancer treatments, including immunotherapy, radiation, surgery, and chemotherapy, are often associated with a range of debilitating side effects such as pain, nausea, loss of appetite, stress, and anxiety, which can severely impact patients' quality of life [6, 7, 8, 9, 10]. Conventional pharmacological interventions are commonly employed to manage these symptoms, yet they may not always provide sufficient relief. As a result, cancer patients explore alternative therapies such as cannabis to help mitigate these symptoms and side effects of cancer treatment [11, 12]. Existing evidence suggests that cannabis may offer palliative benefits, for pain management, antiemetic effects, appetite stimulation, and anxiolytic properties [13, 14, 15, 16]. However, the use patterns and motivations among patients undergoing active cancer treatment remain underexplored.

Emerging literature also indicates that cannabis may interact with cancer treatment modalities, potentially affecting treatment outcomes. Cannabis and cannabinoids have been studied extensively for their antiemetic properties with clinical trials showing that cannabinoids can reduce chemotherapy‐induced nausea and vomiting (CINV) effectively [17]. Some studies suggest that cannabinoids might interact with chemotherapeutic agents, potentially affecting their metabolism and efficacy [18, 19]. Cannabis may be used for postoperative pain management due to its analgesic properties; however, its effects on wound healing and immune function post‐surgery need further exploration [20]. There is limited evidence on how cannabis interacts with anesthetics, with some reports suggesting altered anesthetic requirements and potential complications [21, 22]. Immunotherapy, which harnesses the body's immune system to fight cancer, may have complex interactions with cannabinoids. Understanding these interactions is crucial, as they may influence the efficacy and safety of cancer therapies. For instance, preclinical studies suggest that cannabinoids may modulate immune responses, potentially impacting the effectiveness of immunotherapies. Cannabis can have immunomodulatory effects, which might interfere with immunotherapy. Some studies suggest cannabinoids could potentially suppress the immune system, impacting the efficacy of treatments like checkpoint inhibitors [23]. Animal studies have shown that cannabinoids might influence the tumor microenvironment and immune response, but clinical data are sparse and mixed [24]. Some studies suggest that cannabinoids may have radioprotective effects, potentially protecting normal tissues from radiation damage, which could also mean reduced efficacy of radiation therapy on tumors [25]. Cannabis is also reported to be used for managing side effects of radiation therapy, such as pain and nausea, but its impact on the overall treatment outcome is not well understood [26].

While cannabis shows promise in managing symptoms associated with cancer treatments, its interactions with active cancer therapies remain complex. Investigating the extent and nature of cannabis use among patients undergoing various cancer treatments is essential to developing comprehensive care strategies that optimize therapeutic outcomes while minimizing potential adverse interactions.

This study investigates the patterns of cannabis use among a socio‐demographically diverse group of cancer patients undergoing active treatment at an NCI‐designated cancer center, focusing on sources, dominant cannabinoids, frequency, and reasons for use, whether for symptom management, coping with treatment side effects, or other health‐related purposes. A comprehensive understanding of these dynamics is crucial for healthcare providers to offer informed guidance and support to cancer patients considering cannabis as a complementary therapy. Recognizing the reasons and patterns of cannabis use among patients is vital for informing clinician recommendations and mitigating risks or adverse side effects. Additionally, insights from this study may inform policy decisions and educational initiatives to ensure the safe and effective use of cannabis in oncology settings. By elucidating the sociodemographic factors, sources, and motivations behind cannabis use, this research strives to enhance the understanding of cannabis' role in cancer care, improving patient outcomes and quality of life.

## 2 Materials and Methods

### 2.1 Data Source and Study Sample

Data utilized in this analysis come from phase I of an ongoing two‐phase cross‐sectional study, aiming to collect patient sociodemographic information, cancer‐related data, and cannabis use information to elucidate patterns, reasons, and sources of cannabis use. This study is conducted at an NCI‐designated cancer center, Sylvester Comprehensive Cancer Center (SCCC) at the University of Miami Miller School of Medicine, Miami, Florida.

Included in the analysis were cancer patients aged 18 years or older who visited SCCC in the last 5 years. These included patients undergoing surgical, radiation oncology, comprehensive chemotherapeutic, and immunological treatments, as well as post‐treatment follow‐up visits. Study participants were recruited via phone calls, electronic health portals (such as MyUHealthChart), and direct contact through cancer care teams, with participation encouraged regardless of current or previous cannabis use. The study received approval from the University of Miami Institutional Review Board and the Protocol Review and Monitoring Committee (PRMC) at the SCCC. Informed consent was obtained from the participants, and data collection surveys were administered anonymously via REDCap between October 2021 and June 2023.

### 2.2 Study Measures

Sociodemographic variables including age, sex at birth, race/ethnicity, income, employment status, education level, marital status, healthcare coverage, sexual orientation, and country of birth were collected via self‐report. Likewise, self‐reported data on cancer diagnosis, stage at diagnosis, tumor type, treatment plan, and communication with providers about cannabis were collected from participants.

All participants who received a link to the electronic survey were either in active treatment or within 5 years of initial cancer treatment, confirmed via a REDCap question before the start of the survey. Cancer treatment types were categorized as “Chemotherapy,” “Radiation,” “Immunotherapy,” and/or “Surgery.” Participants selected single or multiple modality treatments depending on their treatment history. For this analysis, we emphasized treatment within the last 6 months of survey completion. This approach was taken to capture recent treatment experiences, ensuring the data accurately represent participants' cannabis use in relation to their current or recent cancer treatments. The cancer stage at diagnosis was self‐reported as “0–4” or other. Due to the anonymous nature of the survey, self‐reported cancer data could not be confirmed via electronic medical records. For this analysis, we included only those under active treatment.

A harmonized questionnaire, developed in collaboration with 11 other NCI‐designated cancer centers, was used to obtain most details about cannabis use (see measure: https://epi.grants.cancer.gov/clinical/nci‐cannabis‐supplement‐core‐measures‐questionnaire.pdf). Cannabis use measures were also self‐reported and included reasons for use, initiation relative to the time of cancer diagnosis, age at first use, last use, frequency of use, source of cannabis, routes of administration, and self‐reported efficacy of cannabis in managing symptoms.

The term “cannabis” in this study encompasses a range of products, including marijuana, cannabis concentrates, edibles, lotions, ointments, tinctures containing cannabis, CBD‐only products, pharmaceutical cannabinoids such as Dronabinol, Nabilone, Marinol, Syndros, Cesamet, and other cannabis‐derived products.

### 2.3 Statistical Analysis

Descriptive statistics were used to summarize the sociodemographic and cancer clinical characteristics of study participants, as well as their cannabis use, both overall and stratified by cannabis use during cancer treatment (yes/no). Active cannabis use was self‐reported by participants; if a participant endorsed cannabis use during treatment, they were classified as “Cannabis Use During Treatment (+CDTX)”; and if a participant self‐reported lifetime cannabis use, but not during treatment, they were classified as “No Cannabis Use During Treatment (−CDTX).” Chi‐squared tests/Fisher's exact tests where appropriate were applied to compare proportions between two groups, +CDTX and −CDTX for patterns, sources, and reasons for cannabis use. Results are reported as means with standard deviation and prevalence (sample sizes and percentages) for numerical and categorical variables, respectively. All analyses were conducted using SAS University Edition with a two‐tailed alpha set to 0.05.

## 3 Results

### 3.1 Sociodemographic Characteristics

A total of 385 participants (mean age = 49.5 years, SD = 15.9) were included in the study, with 158 (41.04%) reporting cannabis use during cancer treatment (Table 1). Participants who used cannabis during treatment were significantly younger compared to those who did not use cannabis during treatment (47.1 years vs. 51.2 years, *p* = 0.01). Of the overall sample, 53.0% were male with no significant difference in cannabis use based on sex at birth (*p* = 0.49). Regarding race/ethnicity, 46.7% were Non‐Hispanic White, Non‐Hispanic Black (6.5%), Hispanic (41.6%), and 5.2% other races/ethnicities. No significant differences were found in cannabis use during treatment among racial/ethnic groups (*p* = 0.76). A majority of participants were born in the United States (US) (73.5%) with a significantly higher proportion of cannabis users during treatment born in the United States (79.1%) compared to non‐users (69.6%, *p* = 0.03). Most participants had healthcare coverage (88.0%). Cannabis use during treatment did not significantly differ based on healthcare coverage status (*p* = 0.54). Regarding employment, 58.5% of participants were employed, 7.5% were unemployed, 21.0% were retired, and 10.4% were disabled. A higher proportion of disabled participants reported cannabis use during treatment (15.8% vs. 6.6%, *p* = 0.04). Income distribution was as follows: 29.4% of participants reported an income of ≤ $34,999, 27.0% had an income between $35,000 and $74,999, and 43.6% reported an income of $75,000 or higher. Educational attainment among participants included 15.0% with high school or less education, 34.0% with technical/some college, 27.0% college graduates, and 23.9% with a post‐graduate degree. Marital status distribution showed that 67.8% were married/living with partners, 13.0% were divorced/separated, 2.3% were widowed, and 16.9% were single/never married. There was no statistically significant difference in cannabis use based on income, education, and marital status (*p* = 0.06).

**TABLE 1:** Sociodemographic characteristics of the study sample by cannabis use during cancer treatment status, *N* = 385.

Characteristics	Overall sample (*N* = 385)	Cannabis use during treatment *n* = 158 (41.04%)	No cannabis use during treatment *n* = 227 (58.96%)	*p*
Age in years				
Mean (SD)	49.5 (15.9)	47.1 (15.5)	51.2 (16.1)	**0.01**
Sex at birth, *n* (%)				0.49
Male	204 (53.0)	87 (55.1)	117 (51.5)	
Female	181 (47.0)	71 (44.9)	110 (48.5)	
Race/ethnicity, *n* (%)				0.76
Non‐Hispanic White	180 (46.7)	70 (44.3)	110 (48.4)	
Non‐Hispanic Black	25 (6.5)	11 (7.0)	14 (6.2)	
Hispanic	160 (41.6)	67 (42.4)	93 (41.0)	
Other	20 (5.2)	10 (6.3)	10 (4.4)	
Born in the US, *n* (%)				**0.03**
Yes	283 (73.5)	125 (79.1)	158 (69.6)	
No	102 (26.5)	33 (20.9)	69 (30.4)	
Healthcare coverage, *n* (%)				0.54
Yes	339 (88.0)	141 (89.2)	198 (87.2)	
No	46 (12.0)	17 (10.8)	29 (12.8)	
Employment status, *n* (%)				**0.04**
Employed	225 (58.5)	86 (54.4)	139 (61.2)	
Unemployed	29 (7.5)	13 (8.2)	16 (7.1)	
Retired	81 (21.0)	29 (18.4)	52 (22.9)	
Disabled	40 (10.4)	25 (15.8)	15 (6.6)	
Other	10 (2.6)	5 (3.2)	5 (2.2)	
Income, *n* (%)				0.65
≤ $34,999	113 (29.4)	43 (27.2)	70 (30.8)	
$35,000–$74,999	104 (27.0)	42 (26.6)	62 (27.3)	
$75,000 or higher	168 (43.6)	73 (46.2)	95 (41.9)	
Education, *n* (%)				0.84
High school or less	58 (15.0)	21 (13.3)	37 (16.3)	
Technical/some college	131 (34.0)	56 (35.4)	75 (33.0)	
College graduate	104 (27.0)	44 (27.8)	60 (26.4)	
Post‐graduate degree	92 (23.9)	37 (23.4)	55 (24.2)	
Marital status, *n* (%)				0.06
Married/living partners	261 (67.8)	108 (68.4)	153 (67.4)	
Divorced/separated	50 (13.0)	16 (10.1)	34 (15.0)	
Widowed	9 (2.3)	1 (0.6)	8 (3.5)	
Single, never married	65 (16.9)	33 (20.9)	32 (14.1)	

*Note: p* < 0.05 is considered statistically significant and indicated in bold.

### 3.2 Cancer Details and Cannabis Use Patterns

**FIGURE 1:**
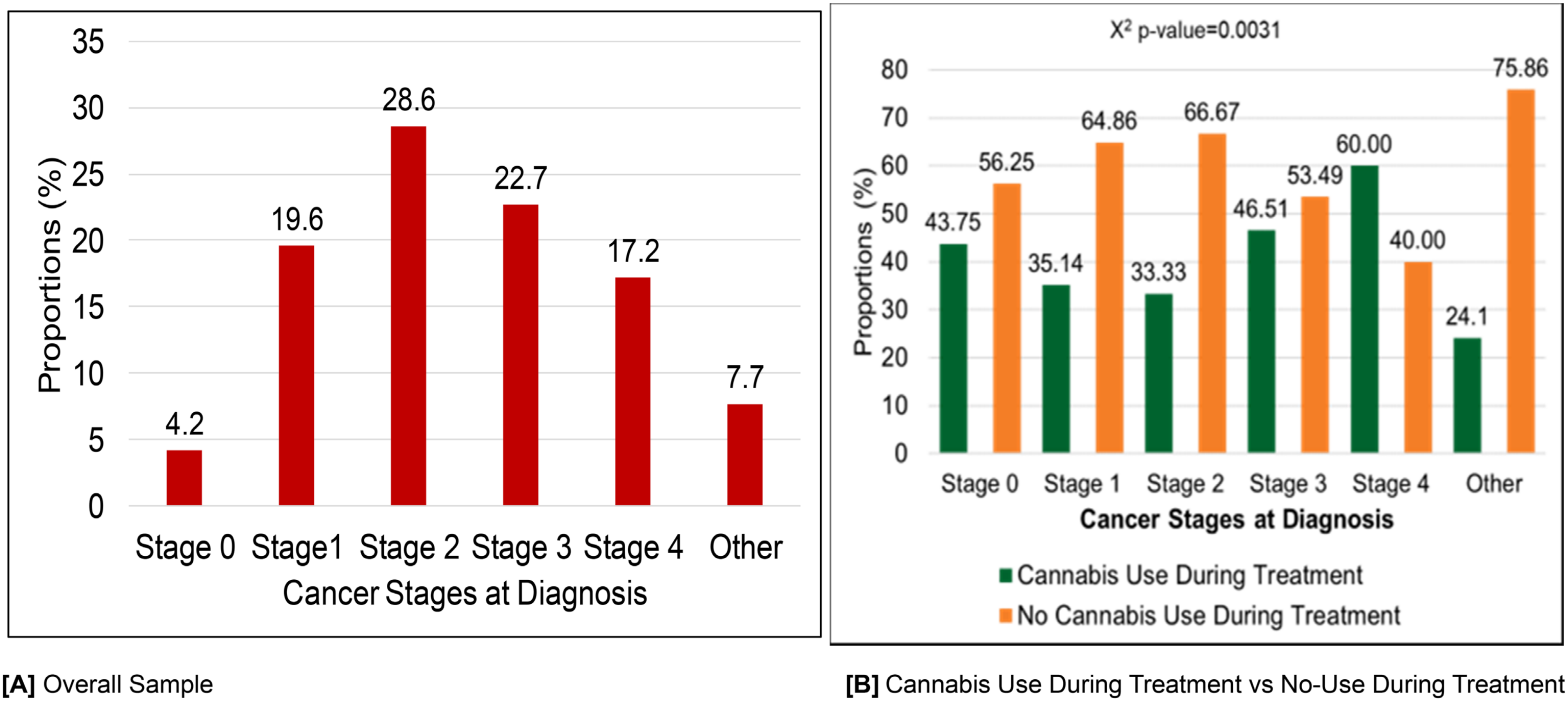
Cannabis use patterns during cancer treatment by cancer stages at diagnosis (*N* = 385). (A) Overall sample. (B) Cannabis use during treatment versus no‐use during treatment. Frequencies of participants' cancer stages at diagnosis were stage 0 (16), stage 1 (75), stage 2 (110), stage 3 (88), stage 4 (66), and others (30).

The distribution of cancer stages at diagnosis among the overall sample is presented in Figure 1A. The most common stage at diagnosis was Stage 2 (28.6%), followed by Stage 3 (22.7%). Figure 1B illustrates the comparison of cannabis use during treatment versus no cannabis use during treatment across different cancer stages at diagnosis. The comparison of cannabis use during treatment versus no‐use during treatment across different cancer stages at diagnosis showed significant differences (*χ* ^2^ *p*‐value = 0.0031). Specifically, Stage 4 patients reported the highest cannabis use during treatment (60.0%), while Stage 2 had the most substantial proportion of non‐users (66.67%).

**FIGURE 2:**
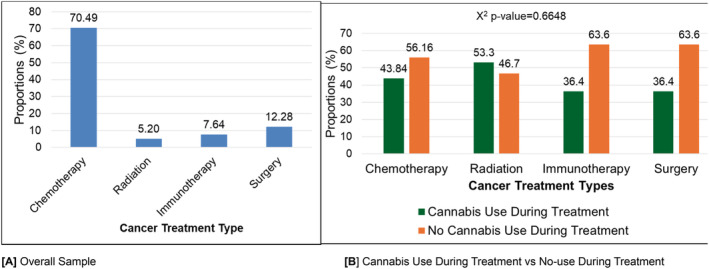
Cannabis use patterns during cancer treatment by cancer treatment types (*N* = 368, missing = 16). (A) Overall sample. (B) Cannabis use during treatment versus no‐use during treatment. Frequencies of participants' cancer treatment type were chemotherapy (271), radiation (20), immunotherapy (430), and surgery (47).

Figure 2 illustrates the patterns of cannabis use during cancer treatment by different treatment modalities. A majority of patients (70.49%) underwent chemotherapy, followed by surgery (12.28%), immunotherapy (7.64%), and radiation (5.20%) (Figure 2A). When comparing cannabis use during treatment across cancer treatment types, no significant differences were observed (*χ* ^2^ *p*‐value = 0.6648) (Figure 2B). During treatment, cannabis use was reported by 43.84% of chemotherapy patients, 53.3% of radiation patients, 36.4% of immunotherapy recipients, and 36.4% of surgery patients.

**FIGURE 3:**
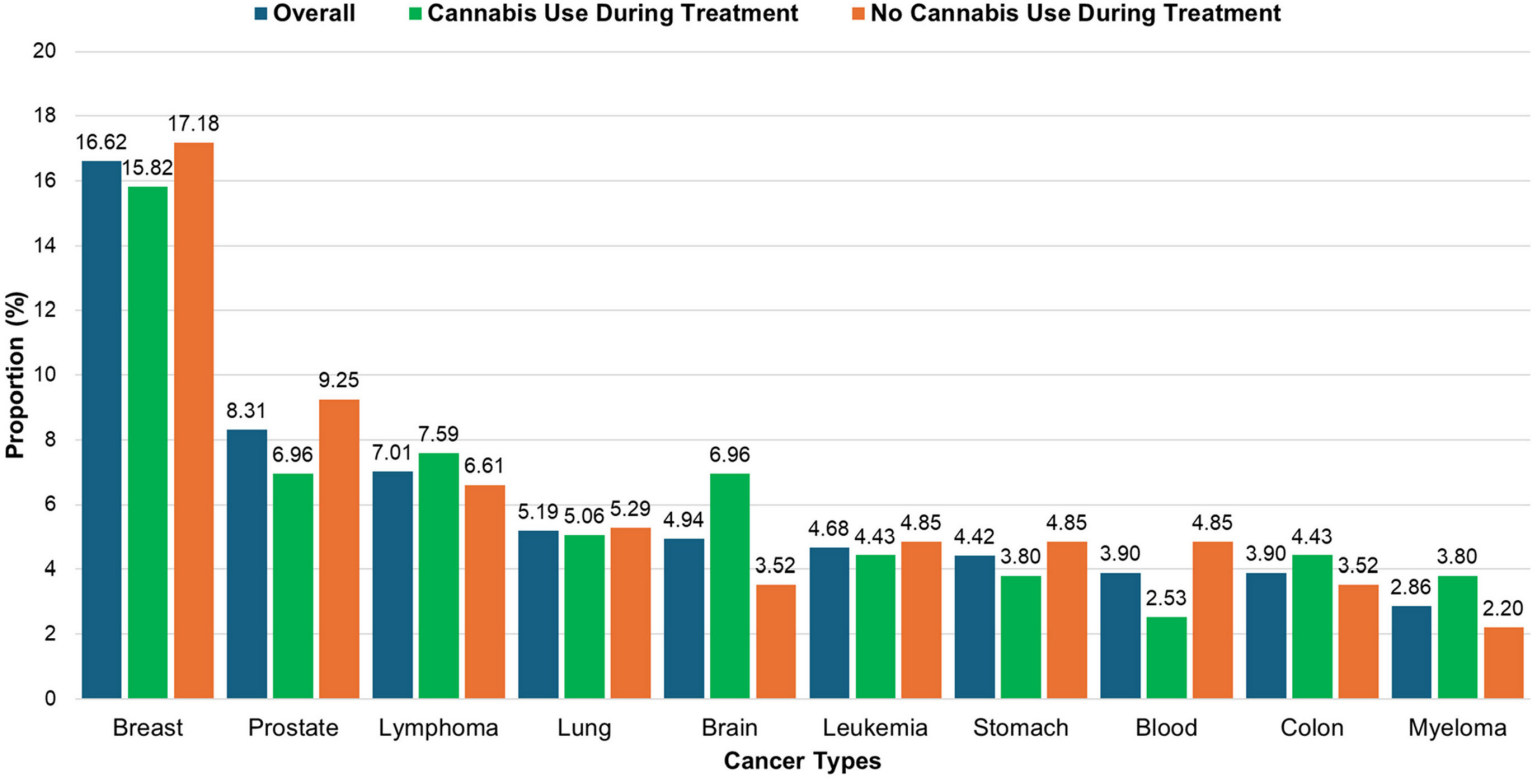
Distribution of cancer types among patient population overall and by cannabis use status during treatment (*N* = 385).

Figure 3 displays the proportion of cannabis use during treatment across different cancer types. Overall, breast cancer accounted for a majority of participants (16.62%) along with the greatest proportion of cannabis users during treatment in the same group (15.82%). The second most prevalent cancer type in our study was prostate cancer (8.31%). Only 6.96% of prostate cancer endorsed consuming cannabis during active cancer treatment. Likewise, 6.96% of participants with brain cancer reported consuming cannabis during treatment. After breast cancer, cannabis use during treatment was higher among lymphoma patients (7.59%). Cannabis use during treatment was lowest in those with blood cancer (2.53%) followed by myeloma and stomach cancer patients (3.80%).

Table 2 presents the cannabis usage patterns, differentiating between participants who used cannabis during cancer treatment and those who did not. A higher proportion of participants (55.1%) reported using cannabis before their cancer diagnosis, with 71.8% of those continuing to use cannabis during treatment, compared to 44.1% of non‐users during treatment (*p* < 0.0001). Dispensaries or medical cannabis stores were the most common sources (48.3%), especially among users during treatment (47.6%). Friends or local dealers provided cannabis to 32.0% of participants overall, with a higher percentage among users during treatment (35.5%) compared to non‐users (24.1%, *p* = 0.05).

**TABLE 2:** Cannabis usage patterns of the study sample.

Characteristics	Overall sample *N* (%)	Cannabis use during cancer treatment	No cannabis use during cancer treatment	*p*
When did you start using cannabis?				**< 0.0001**
Before cancer diagnosis	207 (55.1)	107 (71.8)	100 (44.1)	
After cancer diagnosis	169 (44.9)	42 (28.2)	127 (55.9)	
Where do you get cannabis?				0.05
I grow it myself	2 (1.1)	2 (1.6)	0 (0.0)	
From a friend/local dealer	57 (32.0)	44 (35.5)	13 (24.1)	
Dispensary/medical cannabis store	86 (48.3)	59 (47.6)	27 (50.0)	
Medical co‐op grow	10 (5.6)	4 (3.2)	69 (11.1)	
By prescription from dispensary	14 (7.9)	7 (5.6)	7 (13.0)	
Other	9 (5.1)	8 (6.4)	1 (1.8)	
Since your diagnosis, has a healthcare provider recommended cannabis use?				**< 0.0001**
Yes	94 (25.0)	58 (38.9)	36 (15.9)	
No	282 (75.0)	91 (61.1)	191 (84.1)	
Does your cancer doctor know you consume cannabis?				**< 0.001**
None of my healthcare providers know	33 (18.5)	20 (16.1)	13 (24.1)	
Non‐cancer doctor but another provider knows	67 (37.6)	37 (29.9)	30 (55.5)	
My cancer doctor/team know	78 (43.8)	67 (54.0)	11 (20.4)	
Do you feel comfortable discussing cannabis with your cancer doctor?				0.35
Yes	126 (70.8)	91 (73.4)	35 (64.8)	
No	47 (26.4)	29 (23.4)	18 (33.3)	
I am not currently being treated	5 (2.8)	4 (3.2)	1 (1.9)	
How did you consume cannabis most often?				0.08
Inhale or smoke	78 (54.9)	57 (60.6)	21 (43.7)	
Eat or drink	59 (41.5)	33 (35.1)	26 (54.2)	
Other	5 (3.5)	4 (4.3)	1 (2.1)	
When was the last time you used cannabis?				**< 0.001**
Today	42 (23.6)	34 (27.4)	8 (14.8)	
This week	58 (32.6)	46 (37.1)	12 (22.2)	
This month	34 (19.1)	23 (18.5)	11 (20.4)	
Within the last 6 months	27 (15.2)	14 (11.3)	13 (24.1)	
Within the last year	11 (6.2)	7 (5.6)	4 (7.4)	
Over a year ago	6 (3.4)	0 (0.0)	6 (11.1)	
Why do you consume cannabis?				**0.02**
Depression/to improve mood	62 (34.8)	50 (40.3)	12 (22.2)	
Pain	28 (15.7)	23 (18.5)	5 (9.3)	
Nausea/upset stomach	20 (11.2)	11 (8.9)	9 (16.7)	
For enjoyment/recreational	43 (24.2)	23 (18.5)	20 (37.0)	
Improve appetite	11 (6.2)	7 (5.6)	4 (7.4)	
Help treat cancer	6 (3.4)	3 (2.4)	3 (5.5)	
Cope with cancer	4 (2.3)	4 (2.3)	0 (0.0)	
Deal with stress	4 (2.3)	3 (2.4)	1 (1.8)	

*Note: p* < 0.05 is considered statistically significant and indicated in bold.

Healthcare provider recommendations for cannabis use were more common among users during treatment (38.9%) than non‐users (15.9%), with an overall recommendation of 25.0% (*p* < 0.0001). Disclosure of cannabis use to cancer doctors also varied significantly (*p* < 0.001). While 43.8% of participants had informed their cancer doctor or team, this was more prevalent among users during treatment (54.0%) compared to non‐users (20.4%). Overall, 70.8% of participants felt comfortable discussing cannabis use, with similar proportions among users (73.4%) and non‐users (64.8%) during treatment.

The majority of participants preferred inhaling/smoking cannabis (54.9%), with this method being more common among users during treatment (60.6%). Consumption of cannabis by eating/drinking was the next most common method (41.5%). Recent cannabis use varied significantly (*p* < 0.001), with 23.6% of participants using cannabis on the day of the survey, and a notable proportion using it within the past week (32.4%). Users during treatment reported higher recent use than non‐users during treatment. Participants cited various reasons for cannabis use, with significant differences noted between groups (*p* = 0.02). Improving mood was the most common reason (34.8%), particularly among users during treatment (40.3%). Pain relief (15.7%) and nausea/upset stomach (11.2%) were other common reasons. Notably, 24.2% endorsed using cannabis for enjoyment/recreation, and the proportion was higher in non‐consumers during treatment as compared to consumers during treatment (37.0% vs. 18.5%, *p* = 0.02).

**FIGURE 4:**
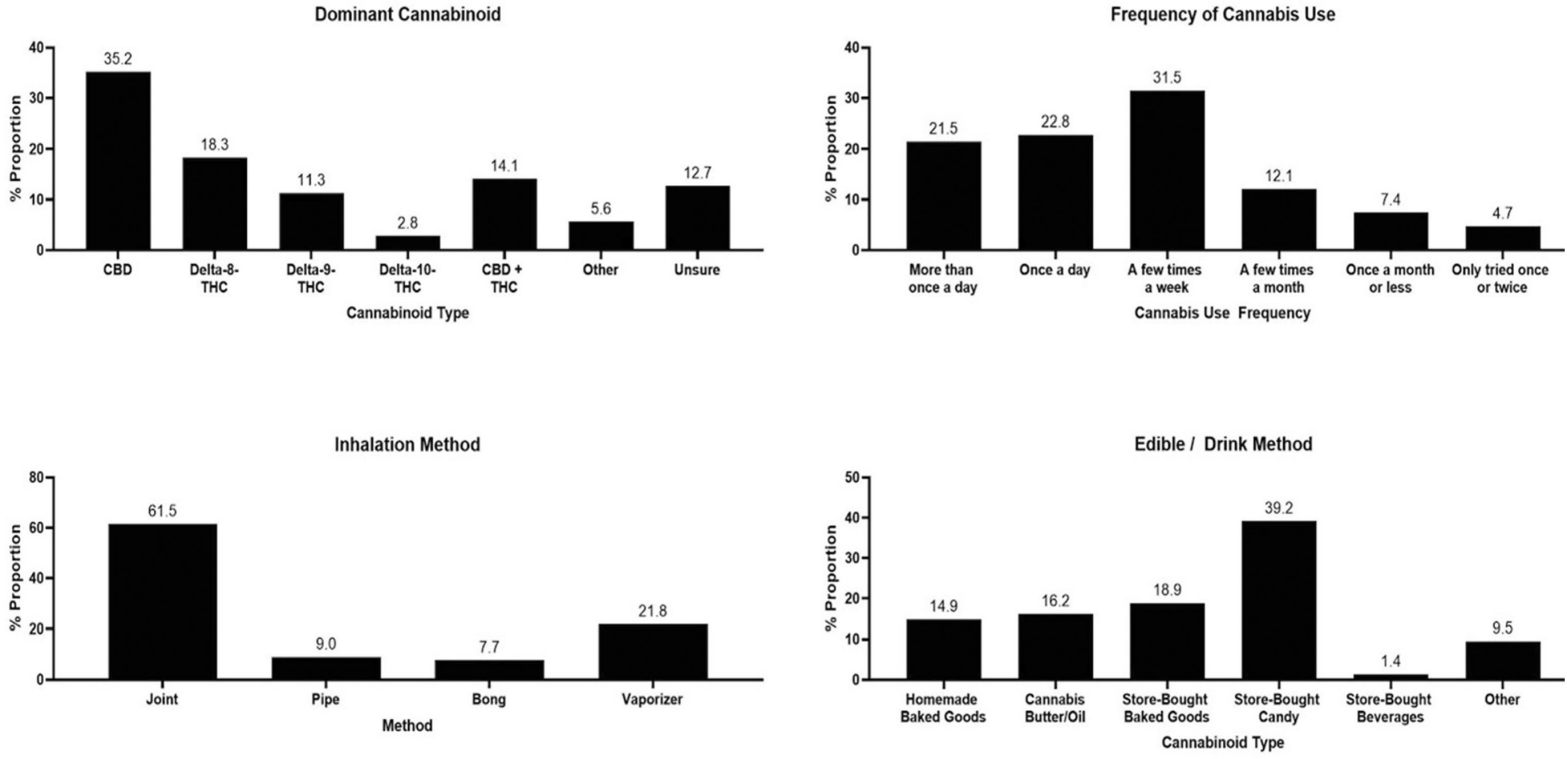
Self‐reported dominant cannabinoid type in their cannabis of use, cannabis use patterns, and ingestion methods among cancer patients who consumed cannabis during cancer treatment (*N* = 158).

Figure 4 illustrates various aspects of cannabis use among cancer patients during their active treatment. CBD was the most dominant cannabinoid within their cannabis of use, self‐reported by 35.2% of participants, followed by Delta‐8‐THC (18.3%), Delta‐9‐THC (11.3%), and Delta‐10‐THC (2.8%). A combination of CBD and THC was endorsed as a dominant cannabinoid by 14.1%, while 12.7% were unsure of the type. Regarding the frequency of cannabis, the most common usage was a few times a week (31.5%), followed by once a day (22.8%), and more than once a day (21.5%). In terms of inhalation methods, joints were the most popular (61.5%), followed by vaporizers (21.8%), pipes (9.0%), and bongs (7.7%). For edible/drink methods, store‐bought candy was the most prevalent (39.2%), followed by store‐bought baked goods (18.9%), cannabis butter/oil (16.2%), homemade baked goods (14.9%), and store‐bought beverages (1.4%).

## 4 Discussion

Our analysis explored cannabis usage patterns among cancer patients undergoing active treatment at a prominent US cancer center. We found that younger, US‐born patients and a larger proportion of disabled participants were more likely to use cannabis during treatment. Notably, stage 4 cancer patients reported the highest proportion of cannabis use, despite stage 2 being the most common cancer stage among respondents. The primary treatment modalities associated with cannabis use were radiation followed by chemotherapy. Surprisingly, over half of the patients began using cannabis before their cancer diagnosis, with a significant number continuing during treatment. Notably, a concerning finding was that many users did not disclose their cannabis use to their cancer care team.

Cannabis was predominantly used to improve mood and manage pain and nausea, with nearly a quarter of patients using it recreationally, especially among non‐users during treatment. Previous research has highlighted self‐reported benefits of cannabis in managing pain, mental health symptoms, and CINV [16, 27, 28, 29, 30]. Studies have also reported that cannabis can help manage chronic pain, which is a common side effect of both chemotherapy and radiation therapy [31, 32]. While cannabinoids, including THC and CBD, have shown potential in symptom management, clinical guidelines remain inadequate due to limited high‐quality evidence [33].

Dispensaries emerged as the primary source of cannabis, reflecting its legalization and normalization, and aligning with broader trends in medical cannabis accessibility enrollment for obtaining therapeutic products [34]. Regarding self‐reported dominant cannabinoid in their cannabis, our findings suggest that patients may prefer CBD over THC, with Delta‐8‐THC potentially being more popular than Delta‐9‐THC due to its milder psychoactive effects and greater availability. Delta‐8‐THC products are commonly sold in various retail outlets, gas stations, and online marketplaces [35, 36, 37]. CBD, a non‐psychoactive compound, is also favored for its potential to alleviate cancer‐related symptoms such as pain nausea and anxiety [38]. Given that a majority of states, including Florida have legalized CBD for medical use, the legality of these cannabis constituents likely influences patient access and preferences [38, 39]. As of early 2021, delta‐8‐THC has rapidly become a popular hemp‐derived product, accessible in most states. However, little is known about its effects or user experiences in medical or recreational contexts [40, 41, 42]. The combination of CBD and THC could be favored for their synergistic benefits [43, 44]. However, studies highlight a complex interplay between the two. In neuropathic pain models, THC and CBD together reduce allodynia with increased potency and fewer side effects [45]. Neuroimaging studies suggest they have opposing effects on brain activation and blood flow in specific regions [46]. Additionally, animal models show that THC can antagonize effects at lower doses but enhance depressant effects at higher doses [47]. This complexity underscores the need for further scientific investigation. In contrast to our findings, a study of 190 cancer survivors at Sheba Medical Center in Israel reported that 46.8% used medical cannabis with an equal THC ratio, while the same proportion were unsure of the dominant cannabinoid. Only 3.7% reported using high‐CBD cannabis, and 2.6% used high THC cannabis [48]. The difference in findings between our study in Florida and Israel could be due to variations in medical cannabis availability, patient education, and prescribing practices. The medical market in Florida has seen a significant rise in the use of high‐CBD formulations, particularly among older adults and palliative care patients. Research indicates that 45% of older adults in Florida utilize CBD‐only products, primarily for chronic pain and musculoskeletal disorders [49]. In Israel, balanced THC products may be more common, with limited access to specialized formulations, leading to more patients using equal‐ratio cannabis or being unsure of the dominant cannabinoid [50, 51]. Cultural attitudes and healthcare guidance may also play a role in these differences [52]. Usage frequency varied with some patients possibly using cannabis daily for consistent symptom relief and others using it intermittently. Inhalation methods, such as joints, might be preferred for their rapid onset, whereas edibles could be valued for their convenience and longer‐lasting effects [53, 54]. The Sheba Medical Center in Israel also reported that the most common way of cannabis administration was smoking followed by vaporizer and ointment [48]. Patient–provider communication about cannabis varied significantly, highlighting the need for improved dialog and integrated supportive care strategies within oncology settings. While healthcare providers historically showed reluctance to discuss or endorse cannabis, evolving attitudes suggest a growing recognition of its potential benefits in supportive cancer care [55, 56, 57].

Our findings corroborate existing literature on the widespread use of cannabis among cancer patients seeking alternative symptom relief beyond conventional treatments [58]. A most recent systematic review conducted among published studies in the US cancer patients and survivors reported that younger age was associated with a greater likelihood of cannabis use [1]. The higher cannabis use among stage 4 cancer patients is primarily due to the need for effective symptom management and palliative care. In alignment with our findings, a study conducted at an NCI‐designated cancer center in Southern California revealed that a higher proportion of patients in stages 3 and 4 considered using cannabis after their cancer diagnosis compared to those who did not [59]. As cancer progresses to advanced stages, patients under active treatment often experience severe pain, nausea, and other debilitating symptoms that significantly impact their quality of life [60]. Similar to our findings, in a study conducted among 1258 patients at Memorial Sloan Kettering Cancer Center in New York, a larger proportion (69%) of cancer patients reported using cannabis before their cancer diagnosis [27]. This suggests that many patients may have prior experience and comfort with cannabis, making them more likely to use it for symptom management during cancer treatment. Interestingly, while 51.7% of participants who used cannabis before their diagnosis continued using it during treatment, approximately 48.3% chose not to. This decision could be influenced by concerns about drug interactions with cancer therapies, guidance from healthcare providers, or changes in personal health management strategies. Further research is needed to explore the factors behind patients' decisions to discontinue cannabis use during treatment.

Our study's findings have important public health and clinical implications. The higher prevalence of cannabis use among younger, US‐born patients and those with disabilities highlights the need for personalized symptom management. Stage 4 cancer patients' high cannabis use underscores the demand for effective palliative care. Significant cannabis use initiation before diagnosis and continuation during treatment indicate potential communication gaps and undisclosed interactions with conventional therapies [33]. Enhanced patient–provider dialog is crucial for informed decision‐making and integrated supportive care. Addressing these issues can improve patients' quality of life, optimize treatment outcomes, and facilitate the integration of cannabinoids into comprehensive cancer care protocols. Clinical guidelines lack robust evidence on cannabinoid efficacy and safety in cancer care, highlighting the need for further research to guide evidence‐based practice.

Our study explored cannabis usage patterns among cancer patients during treatment using a harmonized survey tool developed with 11 NCI‐designated cancer centers. The anonymous cross‐sectional design and high participation rate (70% declined compensation) enhance credibility and provide valuable insights into nationwide trends. However, limitations include small sample size, reliance on self‐reported data without electronic medical records, potential recall bias for treatments up to 5 years prior, and underrepresentation of Non‐Hispanic Black participants, affecting the generalizability of findings given disparities in cancer outcomes among different racial and ethnic groups. Another limitation of the study is the potential overlap between self‐reported reasons for cannabis use, for instance, “To improve mood” and “For enjoyment/recreational,” as these categories may not be fully distinct. The intertwining of emotional well‐being and recreational use may have impacted the interpretation of participants' motivations. Future studies with refined phrasing of response options and larger cohorts would help confirm these findings and further explore the trends observed in this analysis.

## 5 Conclusion

This cross‐sectional analysis of socio‐demographically diverse cancer patients revealed distinct patterns, sources, and motivations for cannabis use during active treatment. Findings highlight widespread cannabis use among cancer patients during treatment, particularly among younger, US‐born individuals and those with advanced disease stages. Notably, many patients initiate cannabis use prior to their cancer diagnosis and continue it during treatment, without disclosing it to healthcare providers. Public health initiatives are warranted to educate patients about the evidence‐based health effects of cannabis. The data gathered from this study should support and prioritize further research to investigate how cannabis and cannabinoids interact with treatment outcomes and potential drug interactions to enhance supportive care strategies in oncology settings.

## Author Contributions

**Amrit Baral:** conceptualization (supporting), formal analysis (supporting), investigation (equal), methodology (equal), project administration (lead), software (equal), visualization (equal), writing – original draft (lead), writing – review and editing (equal). **Bria‐Necole A. Diggs:** funding acquisition (supporting), investigation (supporting), writing – review and editing (equal). **Ranya Marrakchi El Fellah:** investigation (supporting), writing – review and editing (equal). **Connor McCarley:** investigation (supporting), writing – review and editing (equal). **Frank Penedo:** investigation (supporting), validation (supporting), writing – review and editing (equal). **Claudia Martinez:** validation (supporting), writing – review and editing (equal). **Denise C. Vidot:** conceptualization (lead), data curation (lead), formal analysis (lead), funding acquisition (lead), investigation (lead), methodology (equal), project administration (equal), resources (lead), software (equal), supervision (lead), validation (lead), visualization (equal), writing – original draft (equal), writing – review and editing (supporting).

## Conflicts of Interest

The study received approval from the University of Miami Institutional Review Board and the Protocol Review and Monitoring Committee (PRMC) at the Sylvester Comprehensive Cancer Center (SCCC). Informed consent was obtained from the study participants.

## Data Availability

De‐identified data used in this study may be shared with investigators upon request to the principal investigator (Denise C. Vidot, PhD) by e‐mail: dvidot@miami.edu.
